# Supplementary material for: Tumor Microenvironment Alters Chemoresistance of Hepatocellular Carcinoma Through CYP3A4 Metabolic Activity
Source: Front Oncol. 2021 Jun 28;11:662135. doi: 10.3389/fonc.2021.662135 (PMC8273608; doi:10.3389/fonc.2021.662135)
Supplement: Supplementary file 1 [file DataSheet_1.docx]

Supplementary Material

# Supplementary Material – 24 & 48 hours Treatment Findings and Fold Changes

**
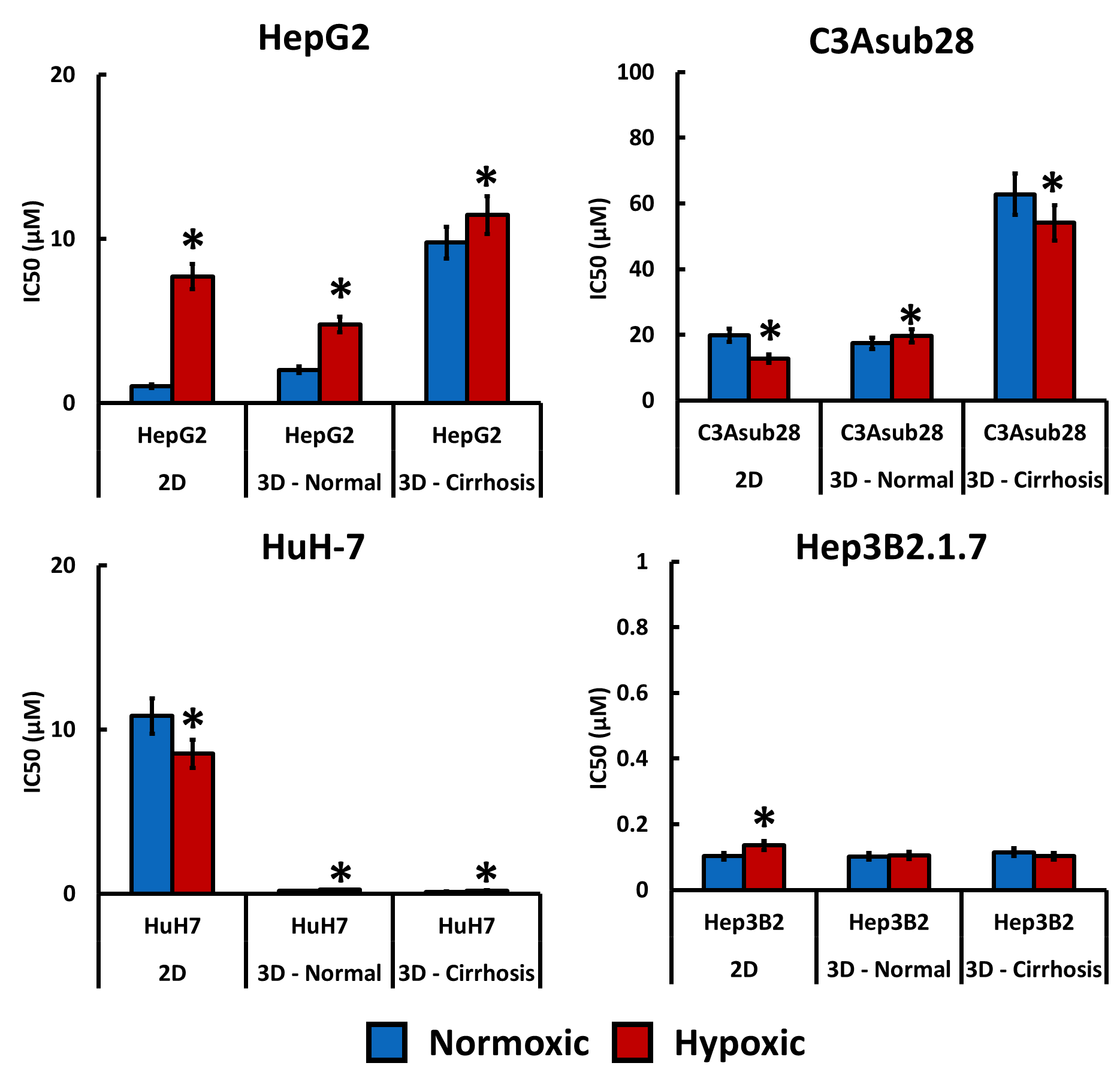
**

**Figure S-I:** Half-maximal inhibitory concentrations (IC50) of HCC cells in different microenvironments. HCC cells have a stiffness and oxygen dependent resistance/sensitivity to 48 hr doxorubicin treatment. Statistical significance was compared to normoxic results. *p<0.05, **p<0.01.

**Figure S-II:** Statistically significant (p<0.05) fold changes in half-maximal inhibitory concentrations (IC50) of HCC cells treated with different concentrations of doxorubicin in varying microenvironments for 24 hours. a) Fold change of IC50 values of 3D-normal stiffness (4 mg/mL collagen) cultured as 2D monolayers comparing normoxic and hypoxic conditions, b) Fold change of 2D-hypoxic cells relative to 2D normoxic cells, c) Fold change of viability values for cells cultured in a 3D-cirrhotic high stiffness microenvironment (7 mg/mL collagen) relative to normal stiffness (4 mg/mL) collagen comparing normoxic and hypoxic conditions, and d) Fold change of viability values of 3D-hypoxic relative to 3D-normoxic conditions comparing normal and cirrhotic stiffness. (Positive values (red) indicate increased viability, negative values (blue) indicate reduced viability, - indicates no significant change determined)

**Figure S-III:** Statistically significant (p<.05) fold changes in half-maximal inhibitory concentrations (IC50) of HCC cells treated with different concentrations of doxorubicin in different microenvironments for 48 hours. a) Fold change of 2D-hypoxic cells relative to 2D normoxic cells b) Fold change of IC50 vales of 3D-normal stiffness (4 mg/mL collagen) to 2D monolayer comparing normoxic and hypoxic conditions c) Fold change of IC50 values of 3D-cirrhotic stiffness (7 mg/mL collagen) relative to normal stiffness (4 mg/mL) collagen comparing normoxic and hypoxic conditions. d) Fold change of IC50 values of 3D-hypoxic relative to 3D-normoxic conditions comparing normal and cirrhotic stiffness. (Positive values (red) indicate increased IC50, negative values (blue) indicate reduced IC50, - indicates no significant change determined)

**Figure S-IV:** a) Fold**-**change in cell viability of HCC cells treated with standard (11 μM) and high (22 μM) sorafenib doses for 24 hours in different 3D microenvironments relative to untreated control. b) Change in cell viability in response to 3D cells in hypoxic conditions relative to 3D cells cultured in normoxic conditions. Fold change was reported from cell viability data (Positive values (red) indicate increased chemoresistance, negative values (blue) indicate reduced chemoresistance, - indicates no significant change determined)

**Figure S-V:** a) Fold change in cell viability of HCC cells treated with standard (11 μM) and high (22 μM) sorafenib doses treated for 48 hours in different 3D microenvironments relative to untreated control. b) Change in viability values of hypoxic condition cells relative to normoxic conditioned. Fold change is reported form cell viability data (Positive values (red) indicate increased chemoresistance, negative values (blue) indicate reduced chemoresistance, - indicates no significant change determined)


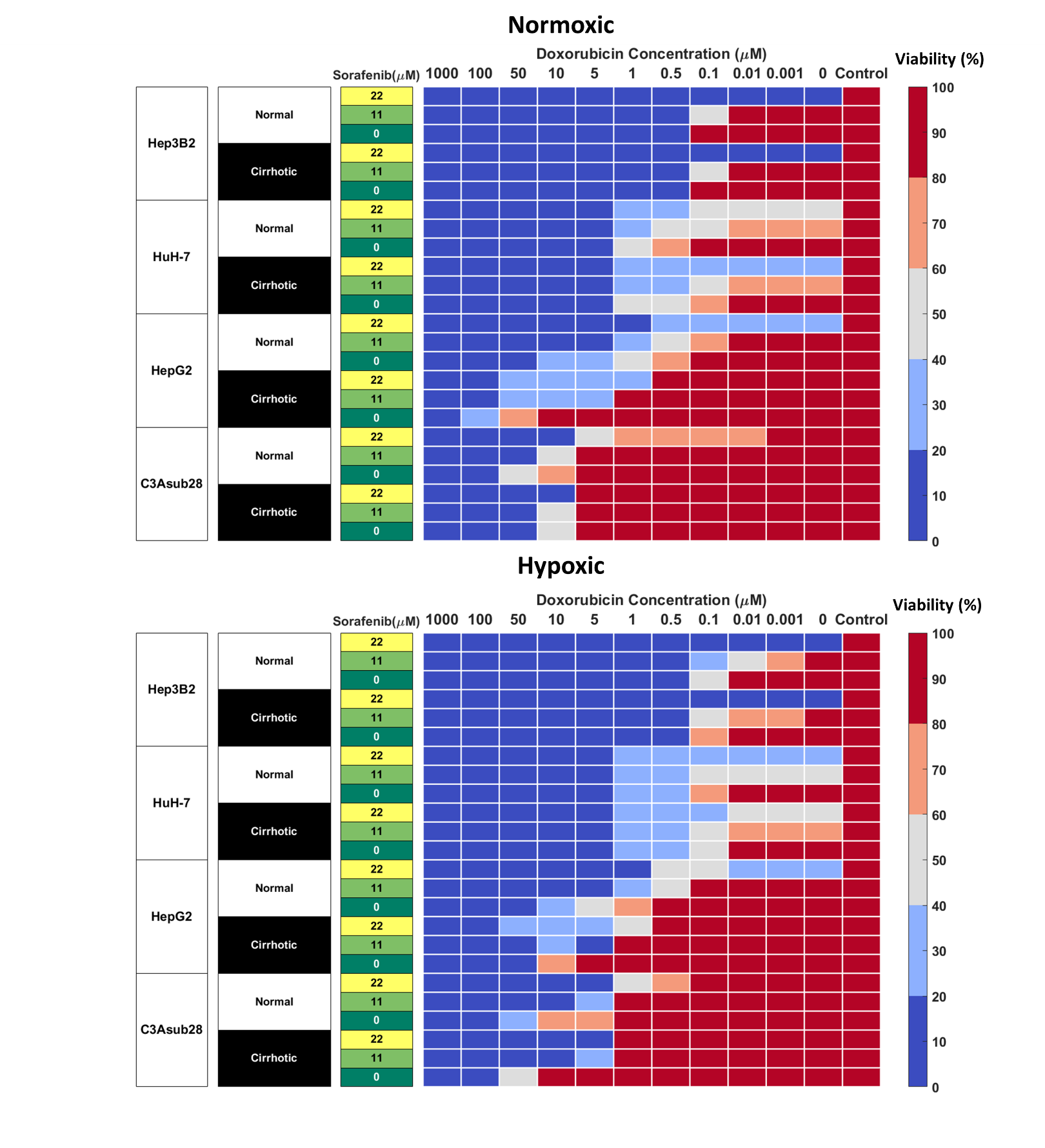


**Figure S-VI:** HCC types show sensitivity to combined sorafenib and doxorubicin treatment for 48 hr. Cells cultured in 3D normal and cirrhotic hydrogels in hypoxic and normoxic conditions. Cell viability was analyzed and plotted as a percentage of untreated control.

**Table S-I:** Modeling parameters for oxygen depletion simulations.

**Table S-II:** Summarized IC50 values of doxorubicin under various doses of sorafenib after 24 hr treatment. n.d.: not detected due to low chemoresistance to sorafenib. Combined doxorubicin and sorafenib treatment findings were statistically compared to doxorubicin alone findings. * p<0.05, ** p<0.01.

**Table S-III:** Summarized IC50 values of doxorubicin under various doses of sorafenib after 48 hr treatment. n.d.: not detected due to low chemoresistance to sorafenib. Combined doxorubicin and sorafenib treatment findings were statistically compared to doxorubicin alone findings. * p<0.05, ** p<0.01.

**References:**

1. Cheema, U., Rong, Z., Kirresh, O., MacRobert, A. J., Vadgama, P., & Brown, R. A. (2012). Oxygen diffusion through collagen scaffolds at defined densities: implications for cell survival in tissue models. Journal of tissue engineering and regenerative medicine, 6(1), 77-84.
2. Weise, F., Fernekorn, U., Hampl, J., Klett, M., & Schober, A. (2013). Analysis and comparison of oxygen consumption of HepG2 cells in a monolayer and three‐dimensional high density cell culture by use of a matrigrid®. Biotechnology and bioengineering, 110(9), 2504-2512.
3. Provin, C., Takano, K., Yoshida, T., Sakai, Y., Fujii, T., & Shirakashi, R. (2009). Low O 2 metabolism of HepG2 cells cultured at high density in a 3D microstructured scaffold. Biomedical microdevices, 11(2), 485-494.
4. Ozkan, A., Ghousifam, N., Hoopes, P. J., Yankeelov, T. E., & Rylander, M. N. (2019). In vitro vascularized liver and tumor tissue microenvironments on a chip for dynamic determination of nanoparticle transport and toxicity. Biotechnology and bioengineering, 116(5), 1201-1219.
